# Supplementary material for: A genome-wide association study reveals the relationship between human genetic variation and the nasal microbiome
Source: Commun Biol. 2024 Jan 30;7:139. doi: 10.1038/s42003-024-05822-5 (PMC10828421; doi:10.1038/s42003-024-05822-5)
Supplement: Supplementary file 2 — Supplementary Information [file 42003_2024_5822_MOESM2_ESM.pdf]

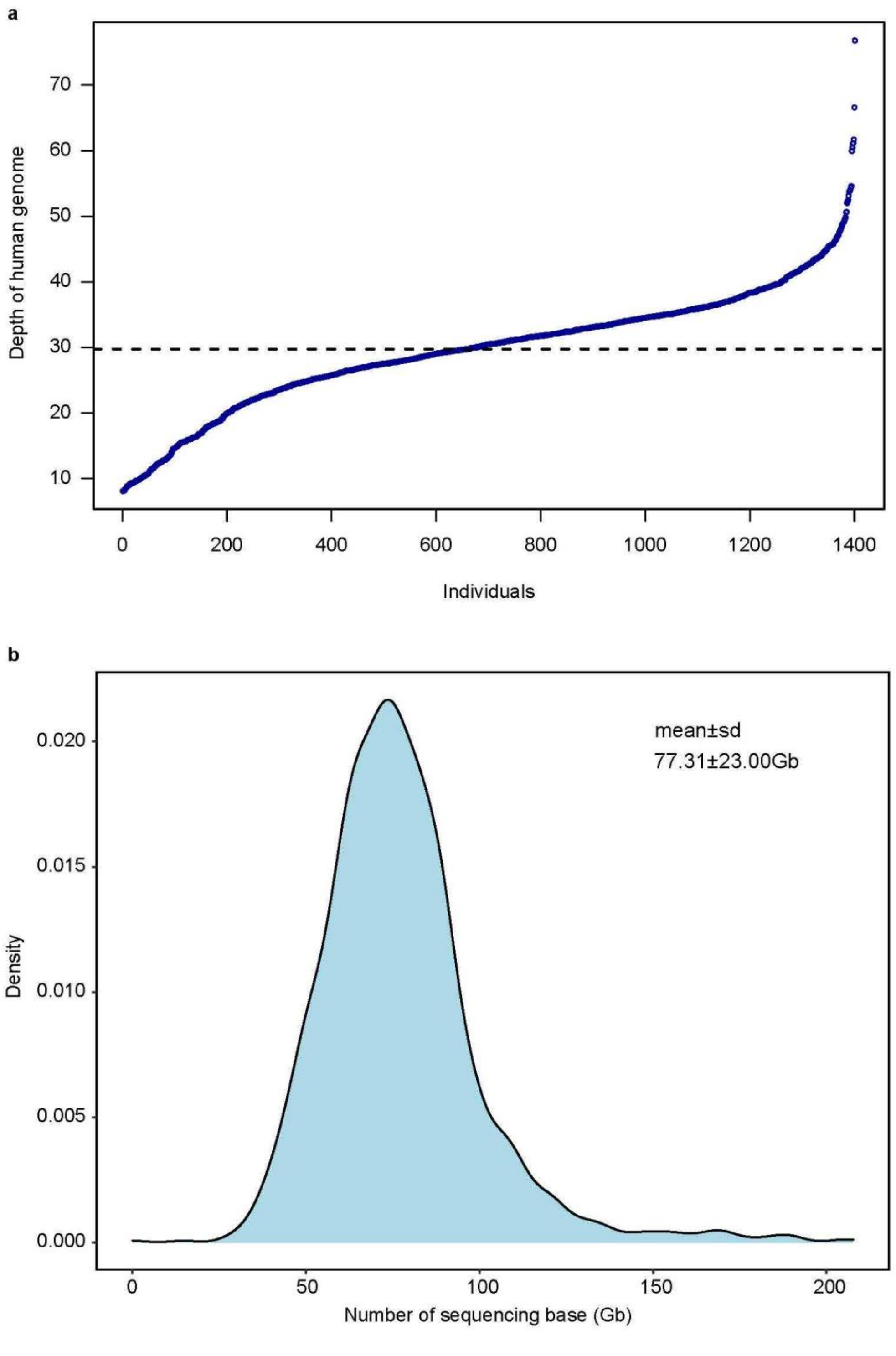

Supplementary Fig. 1. Host genome and metagenome sequencing data production.
a. Depth distribution of 1,401 host genomes by integrating host whole genome sequencing data from blood sample and human-derived reads data extracted from nasal sample. The mean depth is 30× (ranging from 8× to 78×). **b**. Nasal metagenome sequencing at an average of 77.31 ± 23.00 Gb after trimming low quality reads.

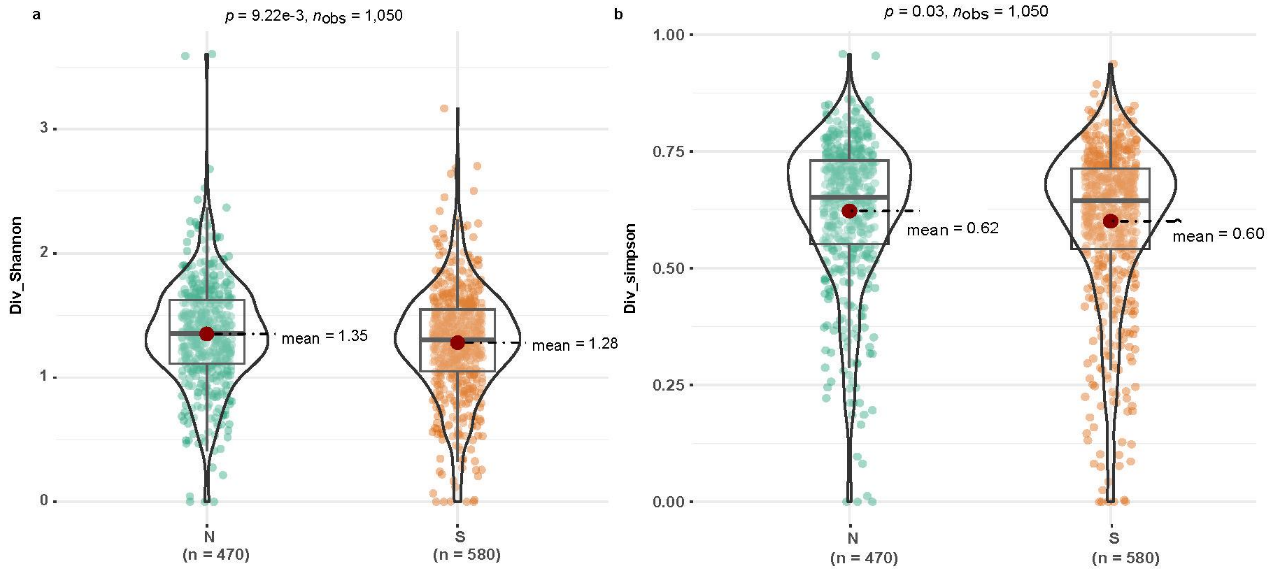

Supplementary Fig. 2. Comparisons of the alpha-diversity of the nasal microbiome between the northern (N) and southern (S) individuals.

Two alpha-diversity indicators, namely Shannon index (**a**) and simpson index (**b**), were plotted between N and S samples, respectively. For all box plots, the central line, box and whiskers represent the median, interquartile range (IQR) and 1.5 times the IQR, respectively. The red dot indicated the mean value. Violin plots represent the distribution density of the data points.

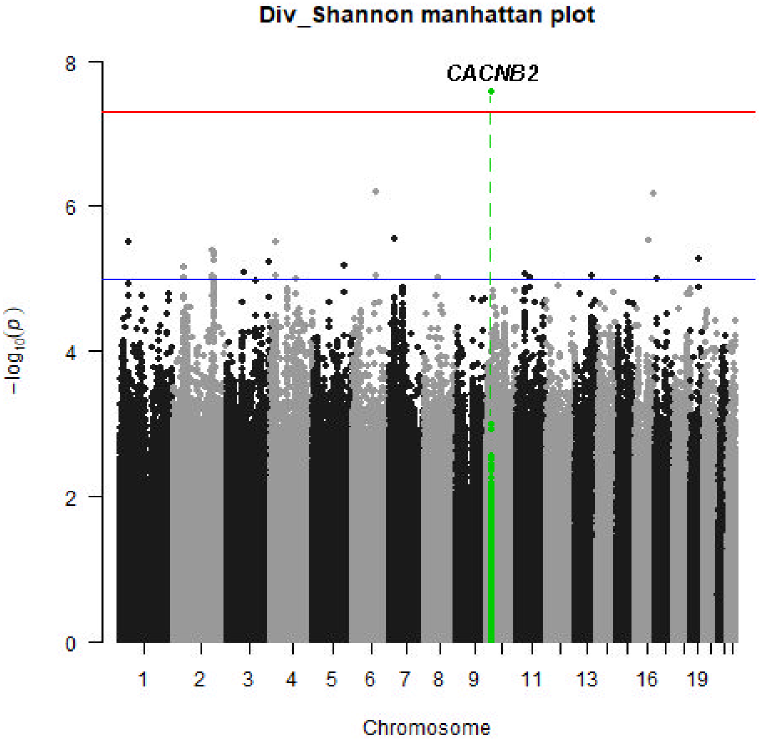

Supplementary Fig. 3. Manhattan plot of nasal microbial alpha-diversity (Shannon index). The red solid line indicates the genome-wide significance level at $P = 5 \times 10^{-8}$ and the blue solid line indicates the suggestive significance level at $P = 10^{-5}$. The top associated signal of gene *CACNB2* was marked in green.

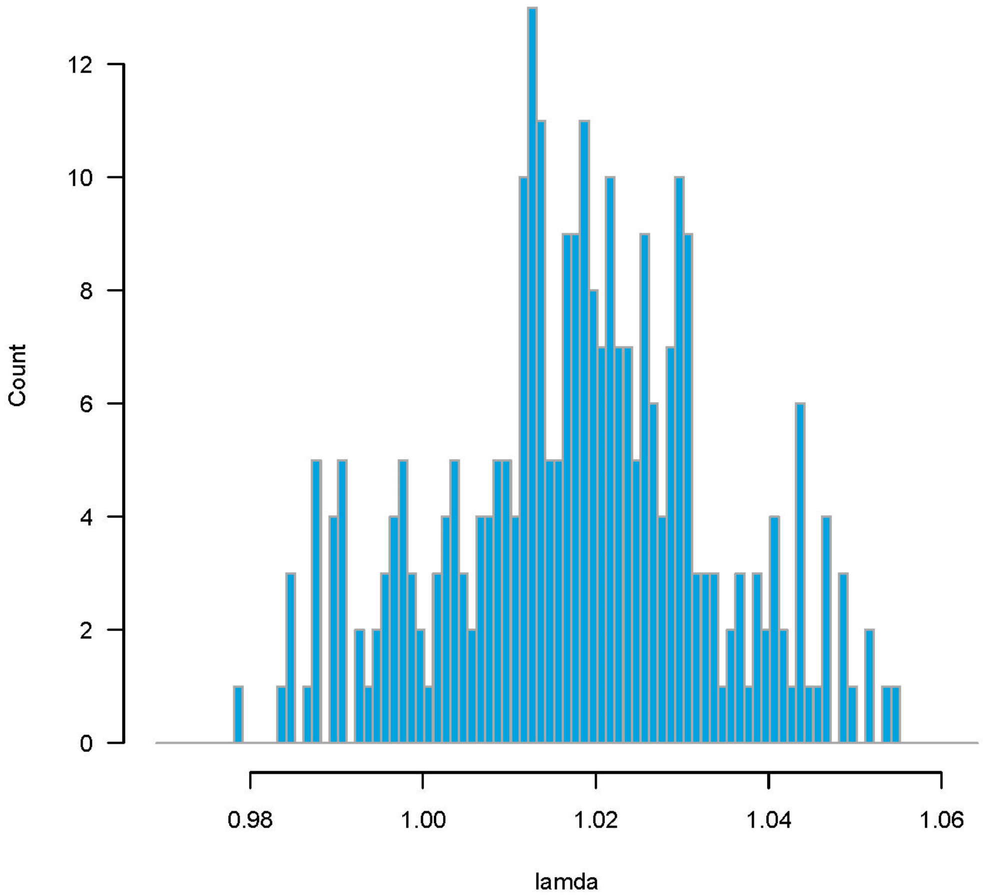

Supplementary Fig. 4. Distribution of the lambda value (λ_{GC}; ranged from 0.979 to 1.054 with a median of 1.012).

There are 293 independent M-GWAS tests performed on the nasal microbial features (86 taxa and 207 functions).

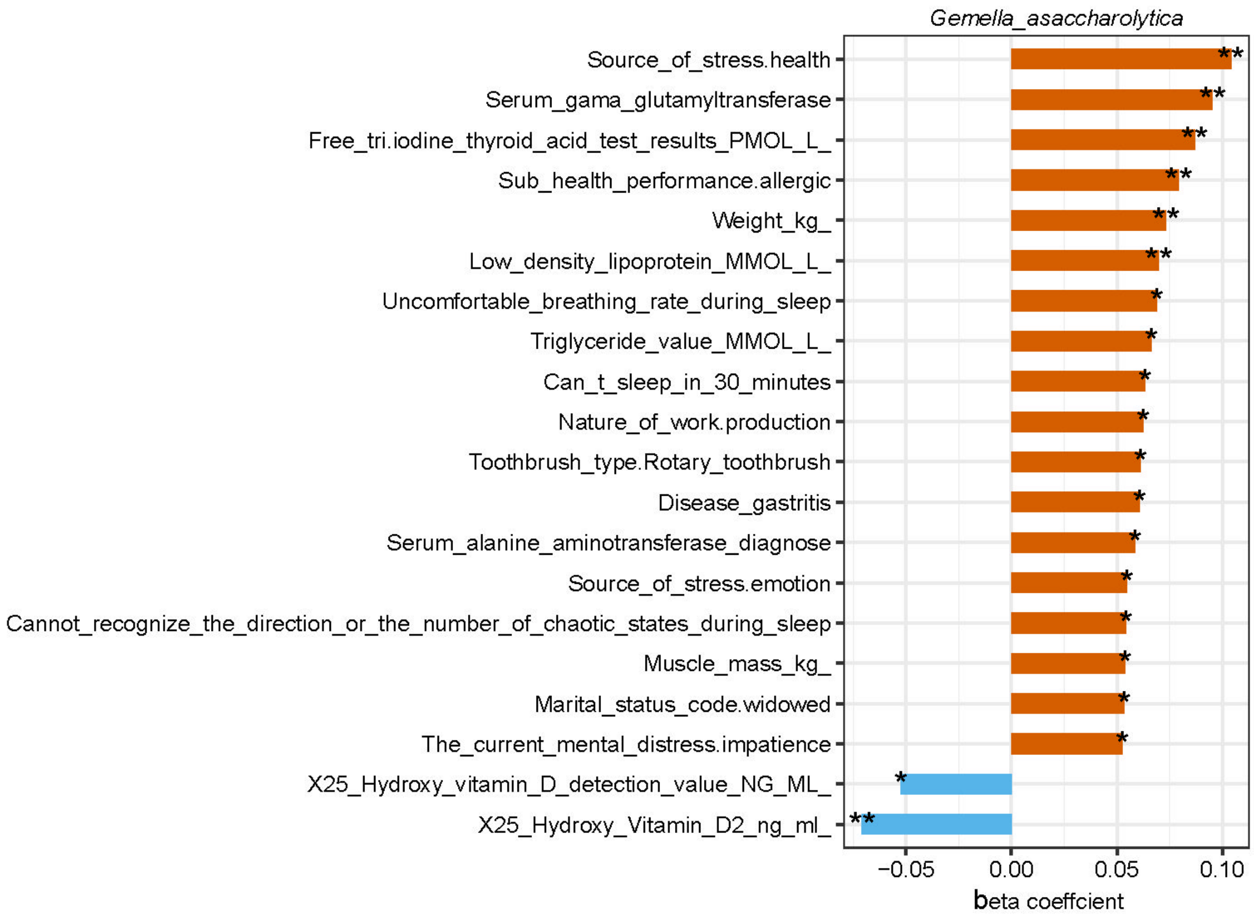

Supplementary Fig. 5. The correlation of species *Gemella asaccharolytica* with host traits in this cohort.

The linear regression model was used with adjusting sex, age and the top four principal components (PC1, PC2, PC3 and PC4). Only correlations with p<0.05 were showed. Significant code: 0.05 * 0.01 ** 0.001.

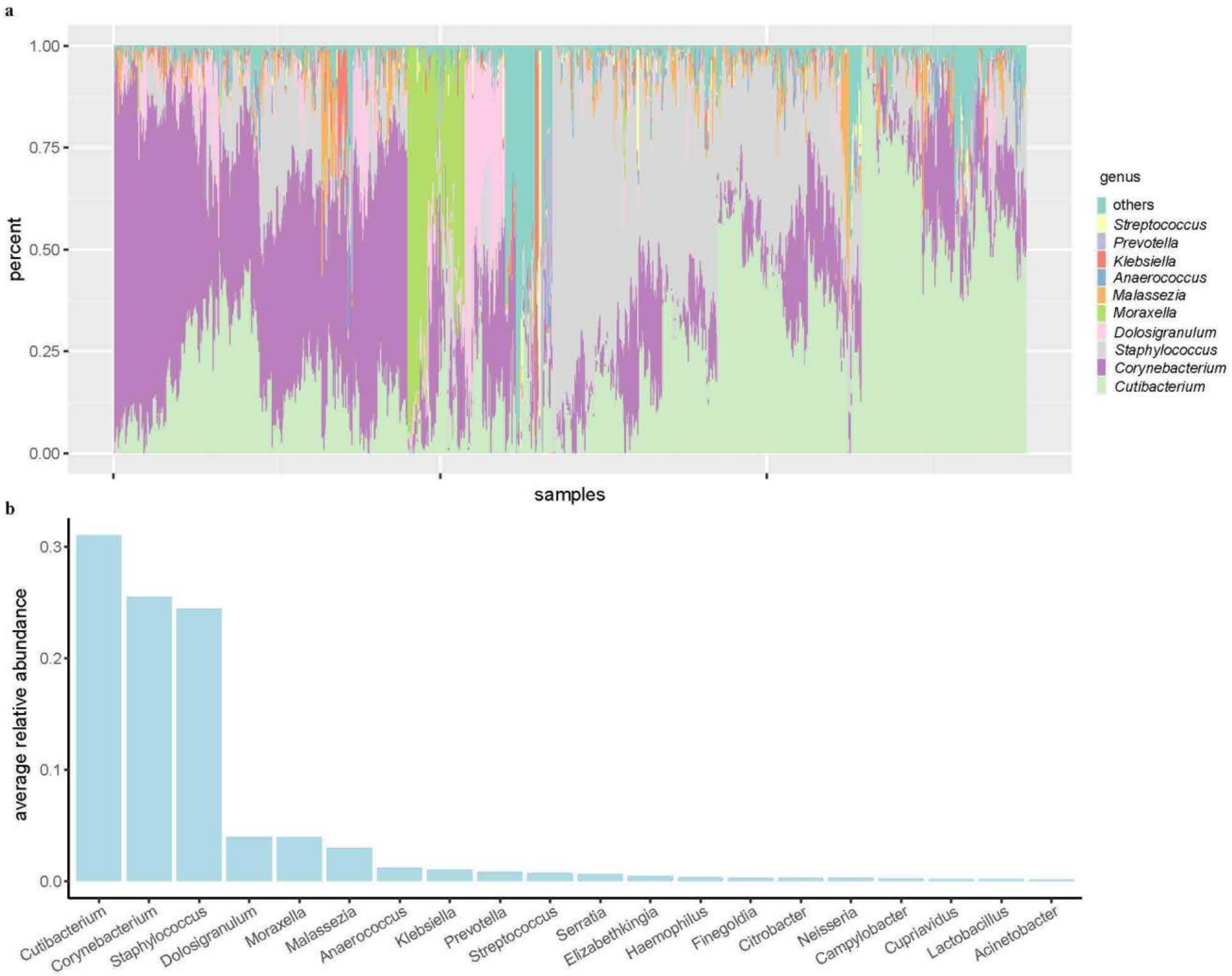

Supplementary Fig. 6. The top genus of the nasal microbiome in this Chinese cohort.
a and **b** showed the top 10 and 20 genus with the highest relative abundances, respectively.

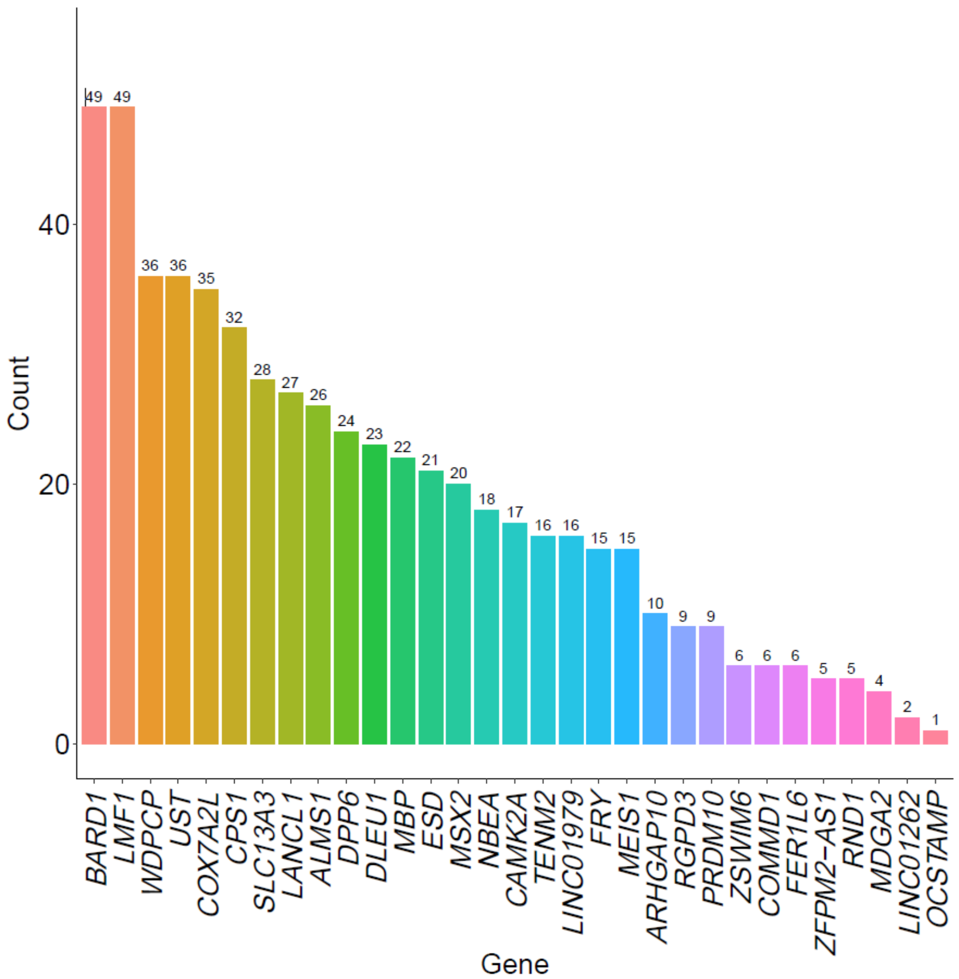

Supplementary Fig. 7. Top genes sorted by the number of tissues in which they are significantly expressed.

26 of the 63 microbiome-associated variants (MAVs) were mapped to 33 genes (intronic or <5KB upstream/downstream). We investigated the expressions of the 33 top genes across 50 tissues and found that *BARD1* (associated with the abundance of genus *Corynebacterium*) and *LMF1* (associated with the abundance of PWY-5686: UMP biosynthesis) were the top two most expressed genes because of cumulative representation across 50 tissues.

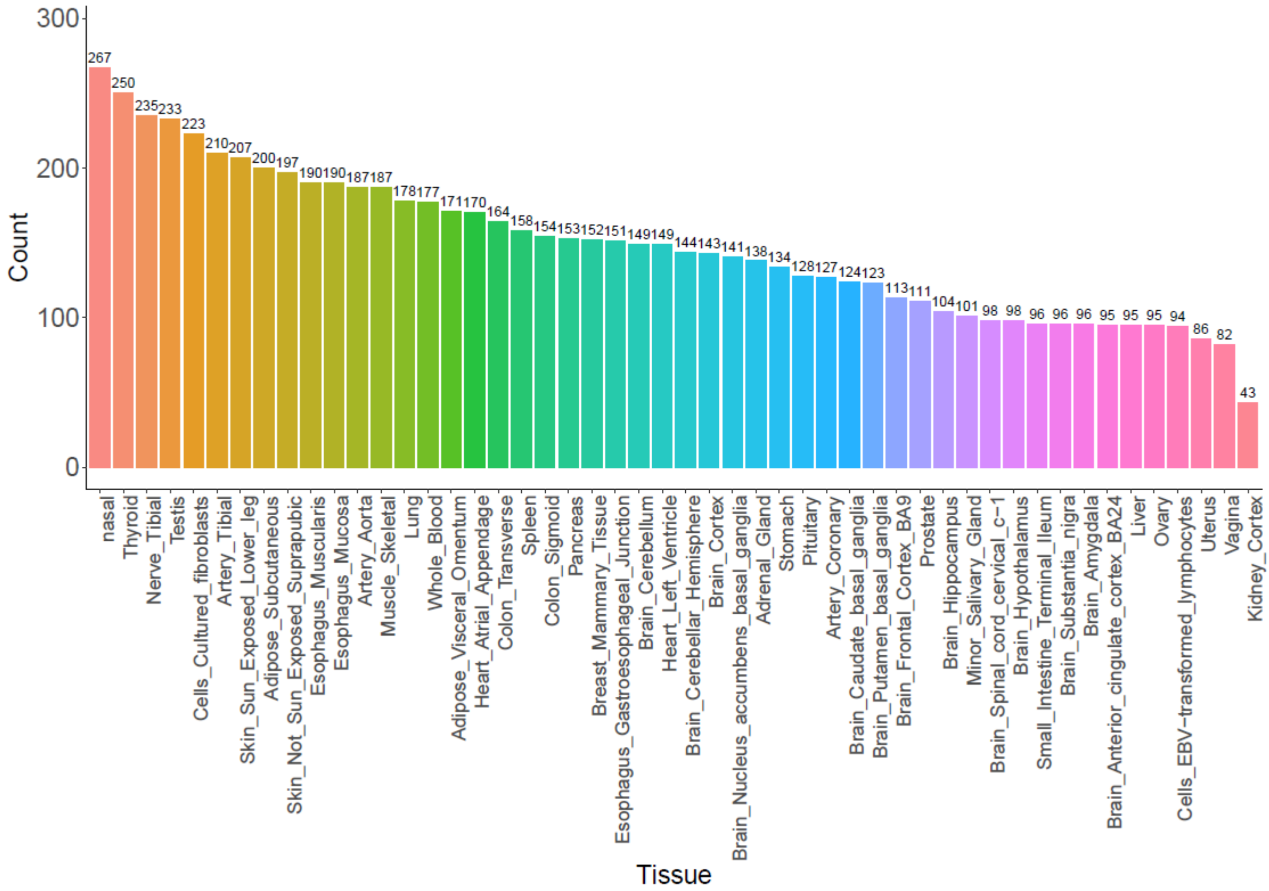

Supplementary Fig. 8. The 413 genes exhibiting suggestively significant nasal MAVs (p<1e-6) were enriched in the nasal airway epithelium and other relevant tissues.

The nasal airway epithelium and other 49 tissue groups from the GTEx database were included for gene expression analysis.

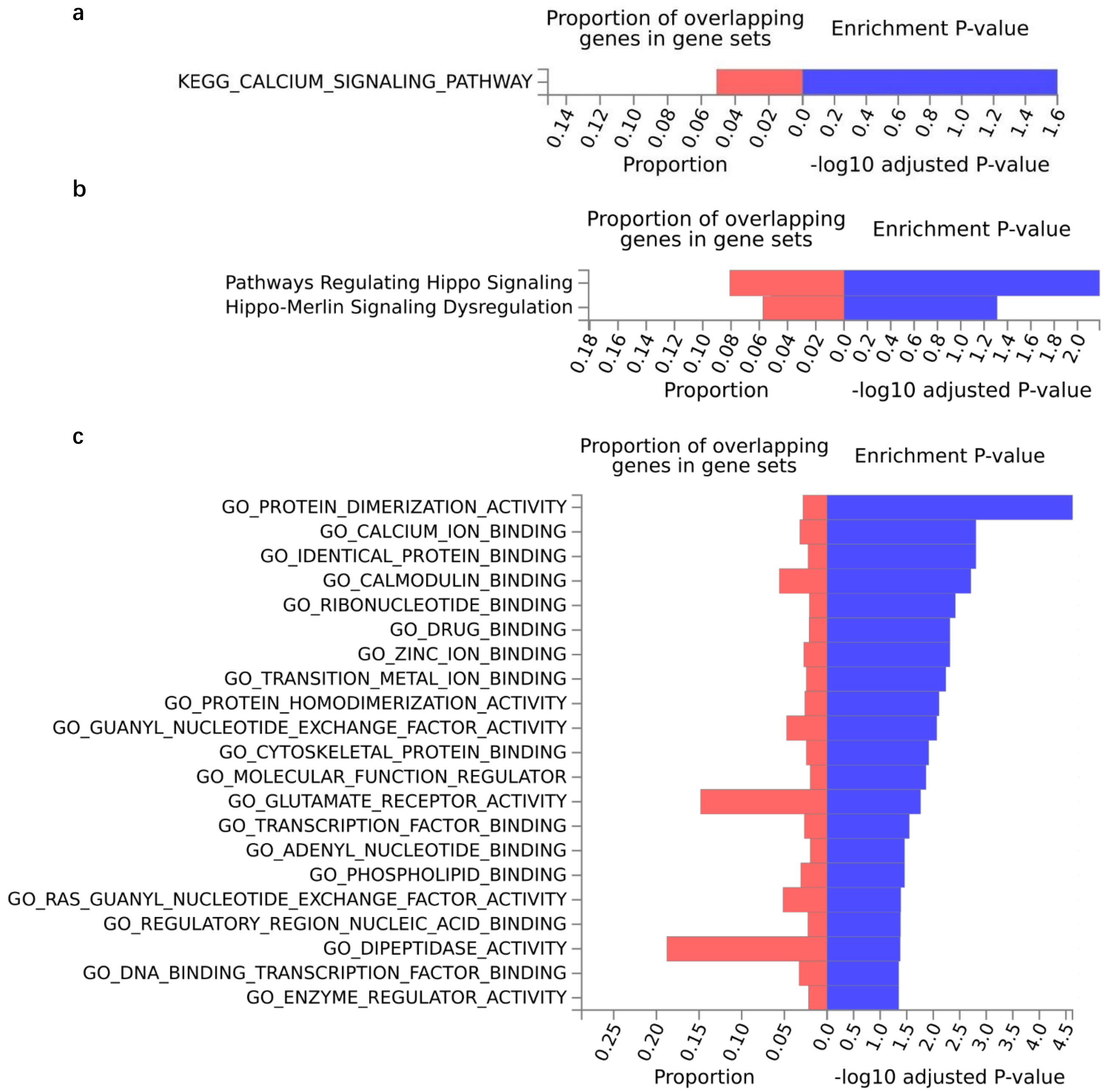

Supplementary Fig. 9. **Gene functional mapping analysis identified enriched pathways using FUMA.**

a. KEGG (MsigDB c2) enrichment results. **b**. WikiPathways enrichment results. **c**. GO molecular functions (MsigDB c5) enrichment results.

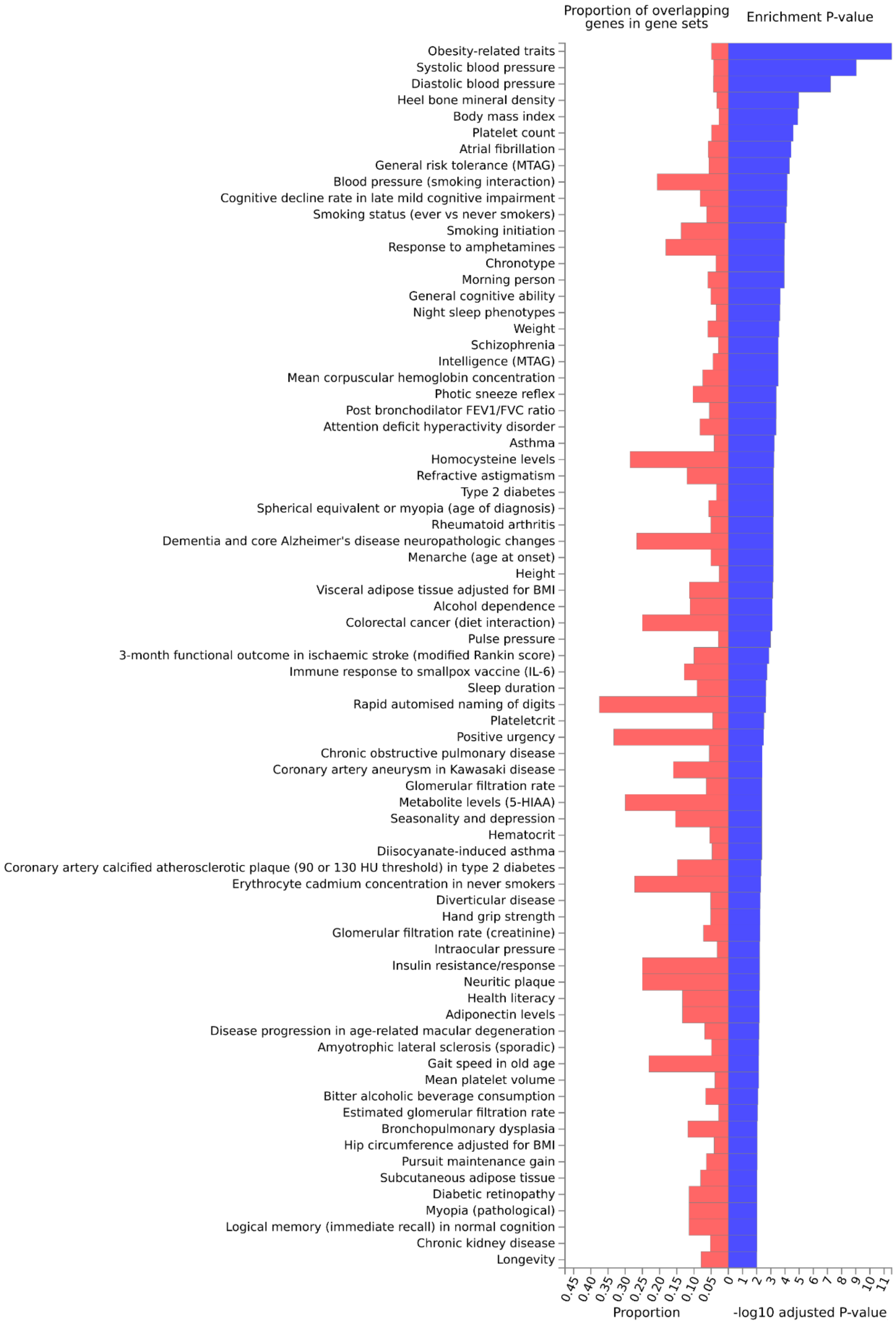

Supplementary Fig. 10. Significantly enriched traits ($p_{adjusted}$ < 0.01) of top signals in the GWAS catalog from nasal M-GWAS analysis.

The significant loci with $p < 10^{-6}$ identified in nasal M-GWAS analysis were mapped to genes based on physical distance within a 5kb window. Mapped genes were further investigated using the GENE2FUNC procedure in FUMA (http://fuma.ctglab.nl/).

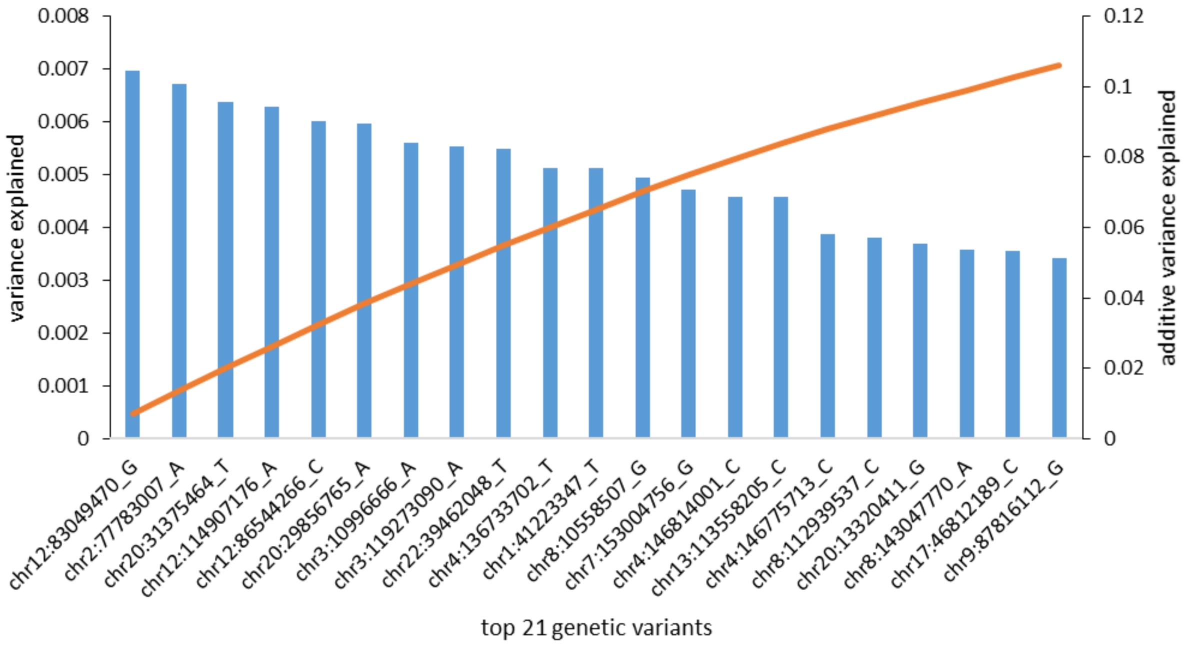

Supplementary Fig. 11. Variance explained by 21 top genetic variants associated with community compositions of nasal microbiome.

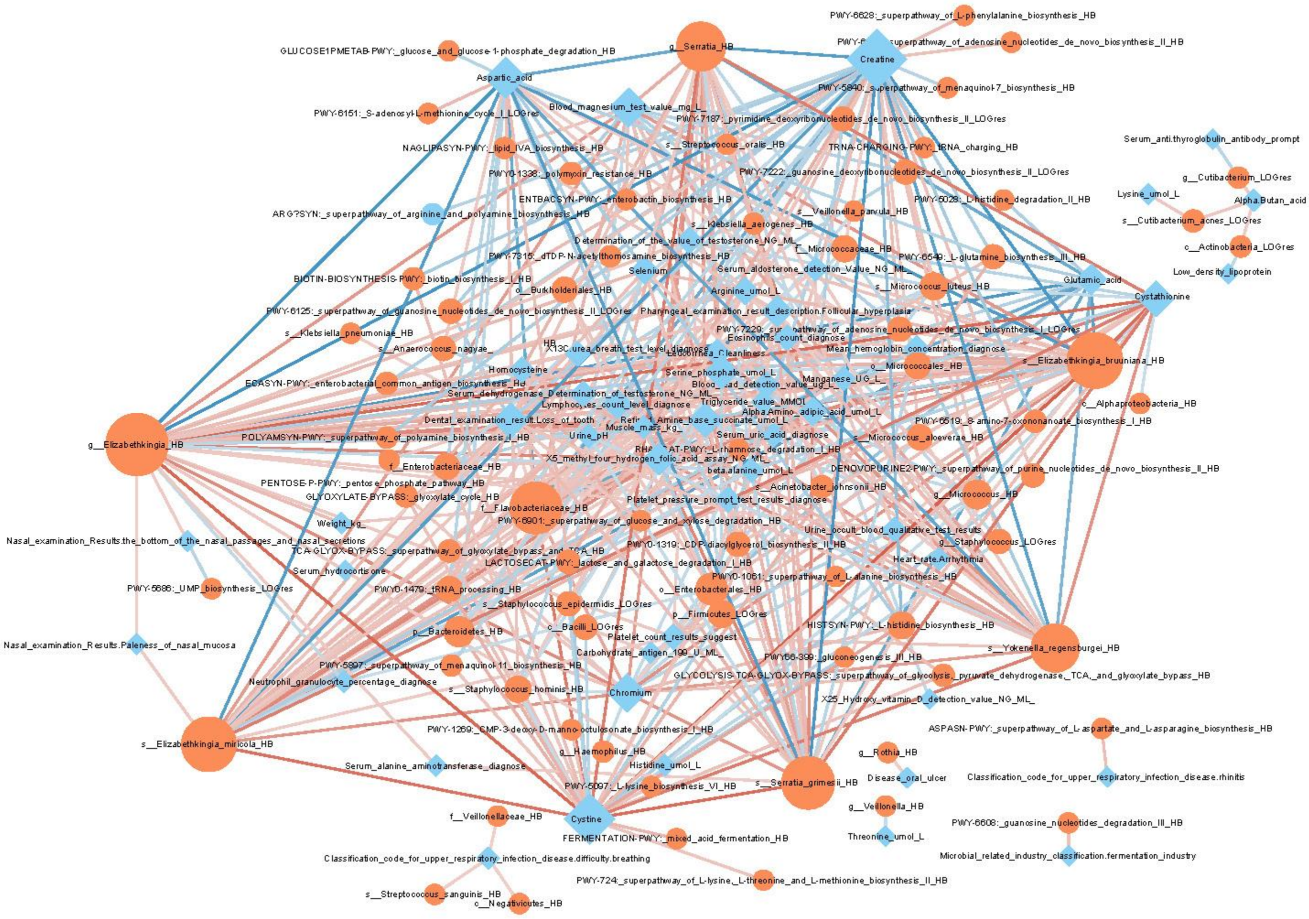

Supplementary Fig. 12. The interaction network represents 402 associations between microbiome features and host traits.

The relationship between host traits (anthropometric traits and blood metabolites) and microbiome features were evaluated by linear regression analysis while adjusted for age and sex. Multiple test correction was performed using p.adjust() function in R (v3.2.5)) with the Benjamini–Hochberg procedure. The 402 significant associations with false discovery rate (FDR) adjusted $P < 0.05$ were showed. Each node represents either a host trait (blue diamonds) or a microbiome feature (orange circles). Each edge is an association between one host trait and one microbiome feature. Node size is directly proportional to the number of other nodes associated with it. Red edges correspond to positive correlations and blue edges to negative correlations. The 402 associations were listed in the Supplementary Table 11.

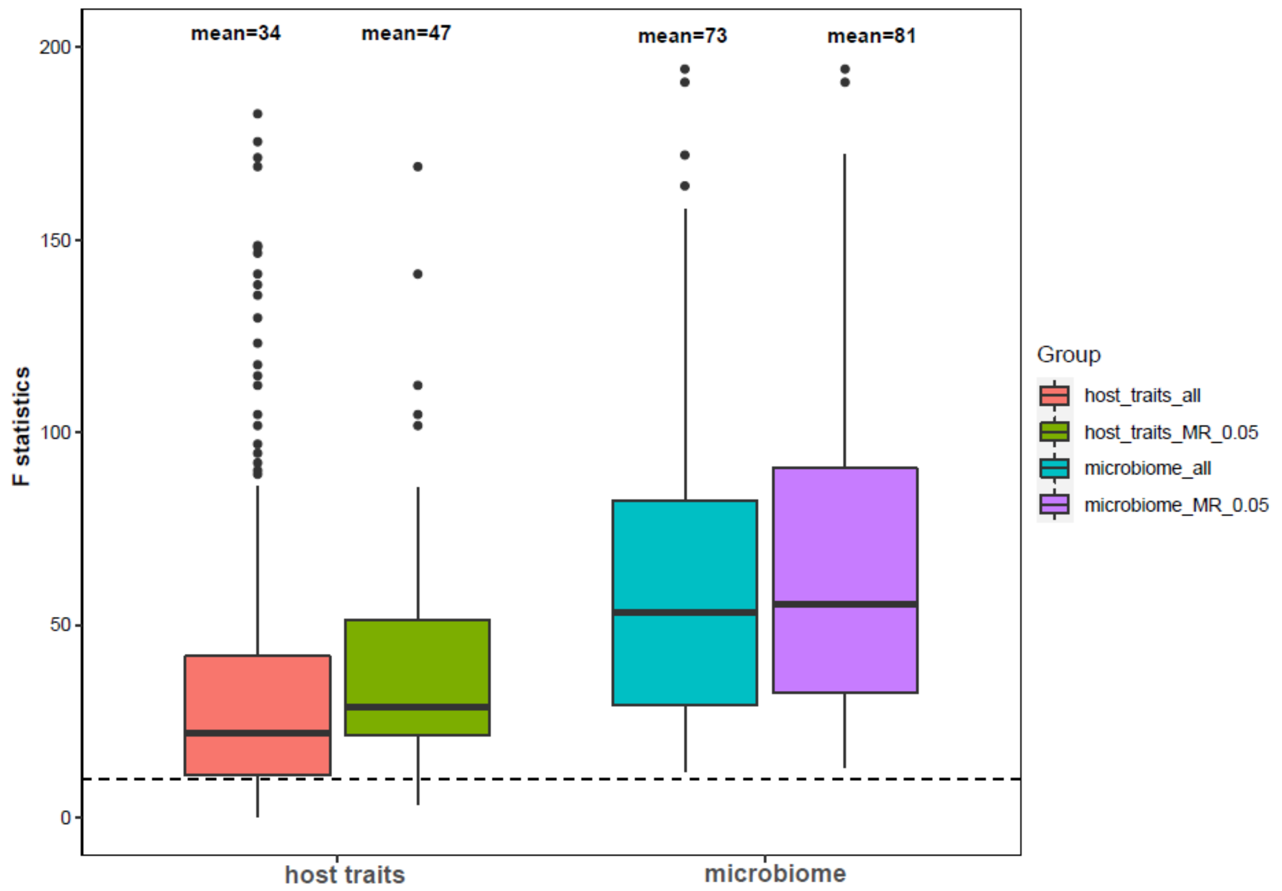

Supplementary Fig. 13. Distribution of the instrumental F statistics for the nasal microbiome and host traits (mainly metabolites) in this study.

For each nasal microbiota, we first selected genetic instruments using $p < 1 \times 10^{-6}$. Then, we tested the strengths of these instruments and the mean instrumental F statistics were all greater than 10 (the dotted line), indicating a strong instrumental strength. For all box plots, the central line, box and whiskers represent the median, interquartile range (IQR) and 1.5 times the IQR, respectively.

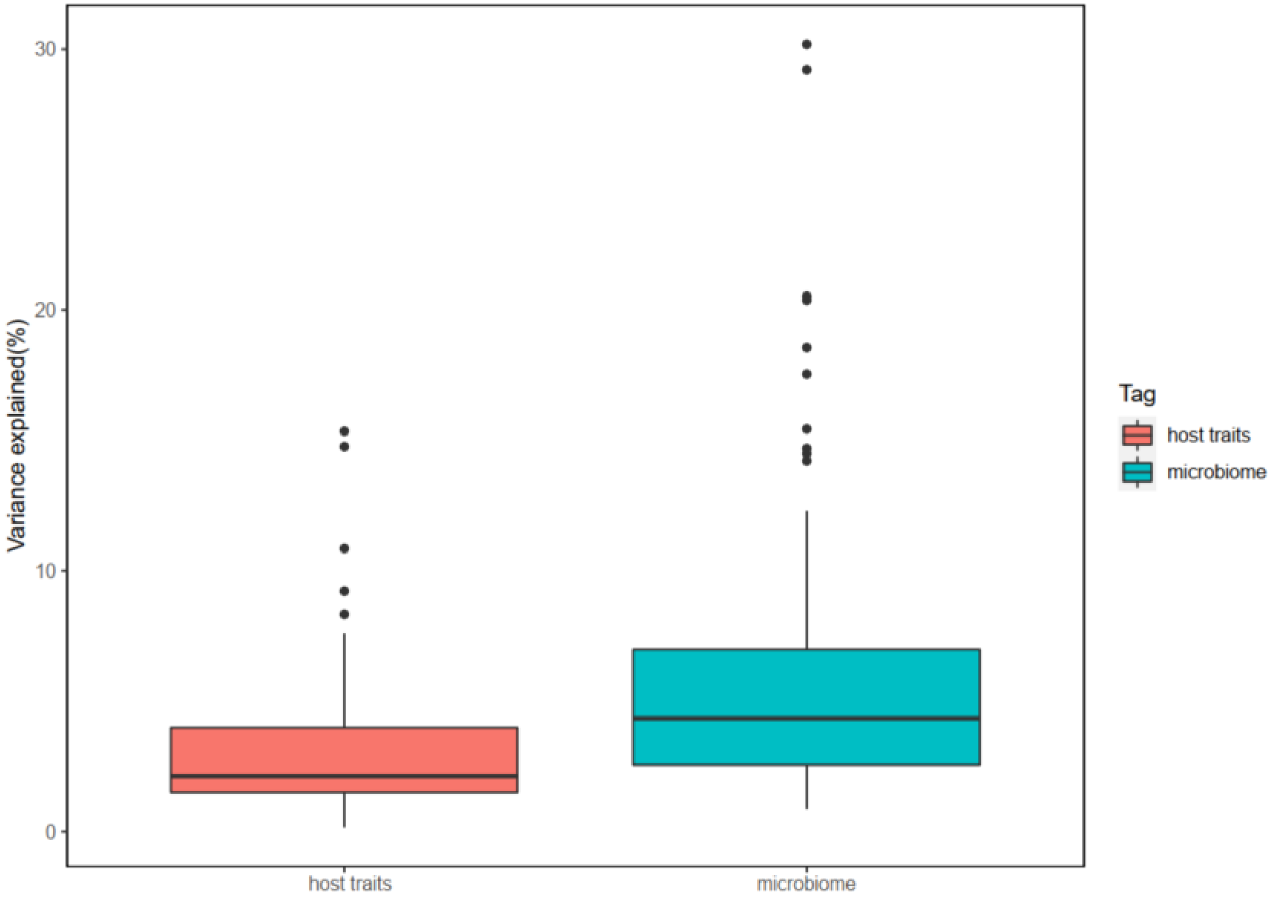

Supplementary Fig. 14. Distribution of variance explained for microbiome features in this study.

We showed the distribution of variance explained of microbiome features and host traits by genetic predictors selected in whole-genome wide association analysis at *P*-value threshold 1e-6 (boxplot). For all box plots, the central line, box and whiskers represent the median, interquartile range (IQR) and 1.5 times the IQR, respectively.

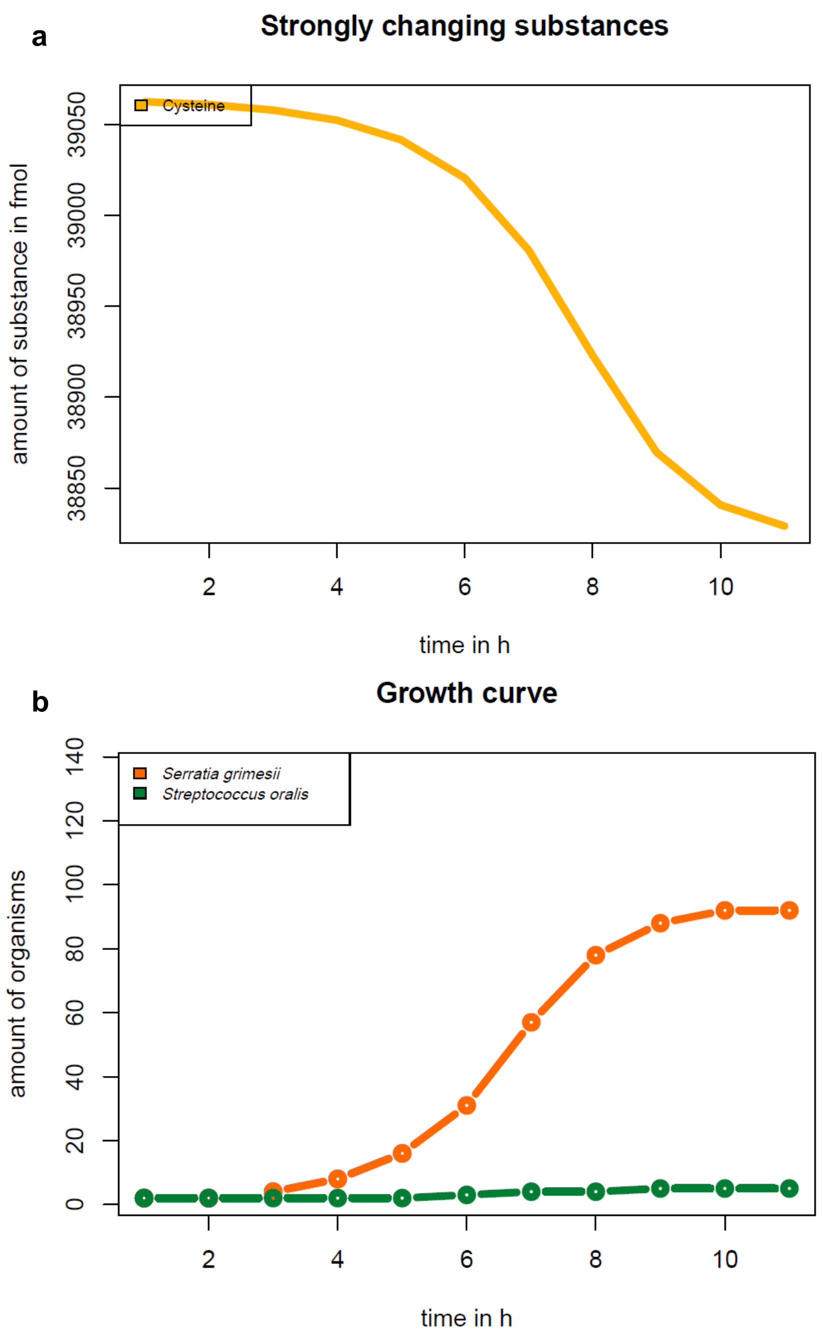

Supplementary Fig. 15. The cysteine consumption and microbial growth were predicted by the gapseq model.

a. the cysteine metabolic consumption changed over time(h). **b**. the growth curve of *Serratia grimesii* compared to *Streptococcus oralis* under the cysteine environment as predicted in the model.

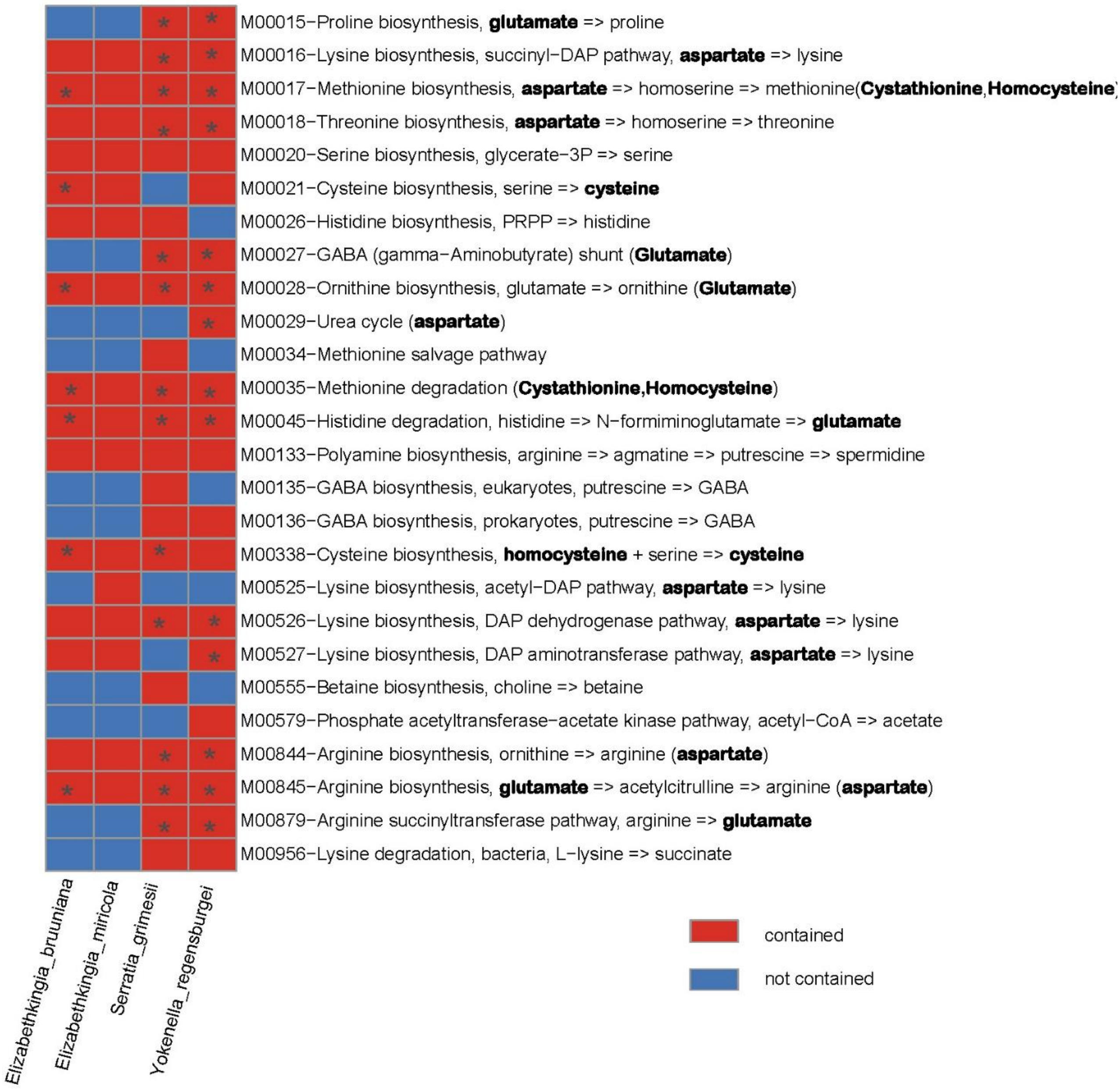

Supplementary Fig. 16. Functional modules of the four nasal bacteria identified by MR associated with serum amino acids.

Genome-wide functional annotation was done by the eggNOG mapper (http://eggnog5.embl.de/). The red cell represented the genome of the microbiome contained this module or enzyme. The blue cell represented the genome of the microbiome didn't contain this module or enzyme. The "*" indicated the nasal microbiome listed were also associated with the module or enzyme involving the corresponding serum amino acids in the MR analysis.

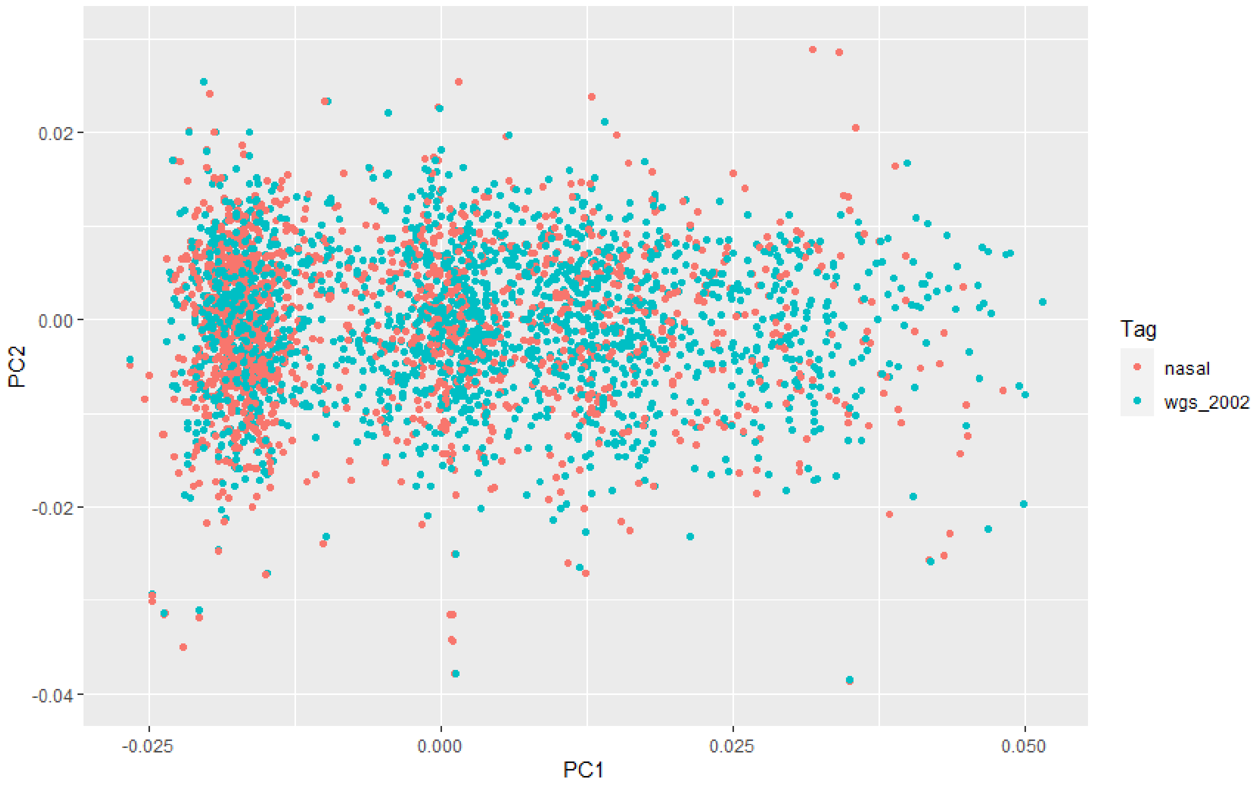

Supplementary Fig. 17. PCA analysis showed no population stratification between 1401 integrated WGS samples in this study and 2002 blood WGS samples as reported previously.

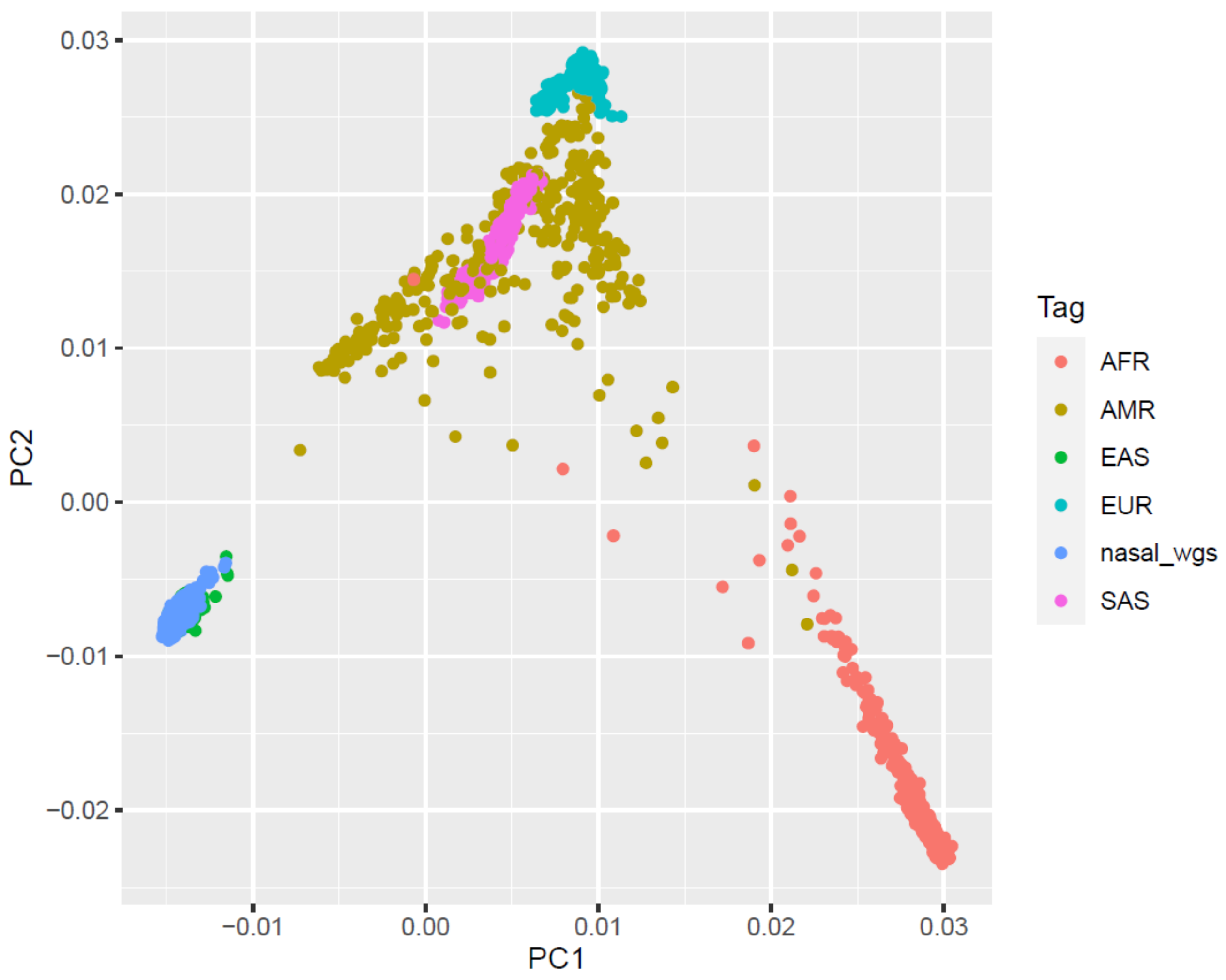

Supplementary Fig. 18. PCA analysis showed all individuals in this cohort (Chinese; nasal_wgs) clustered into the EAS (East Asian) group and significantly separated from the AFR and EUR populations.

The data were from this study (nasal_wgs) and 1000genome phase 3 datasets (AFR, AMR, EAS, EUR, and SAS).

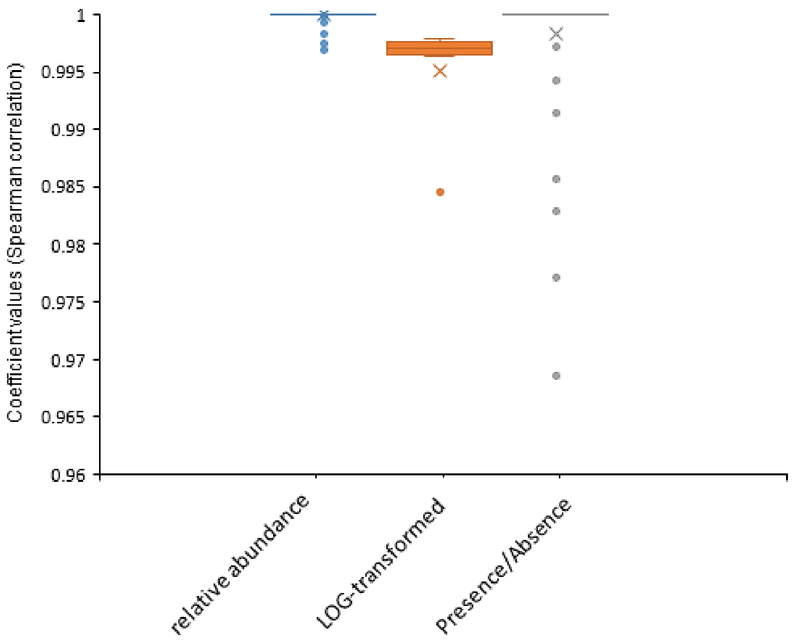

Supplementary Fig. 19. **The Spearman correlations of two different profiles in bacteria taxa quantification.**

We constructed two MetaPhlAn3 profiles, one using bacteria and fungi together and the other only using bacteria as microbial community, respectively. The comparisons involved in the raw relative abundances of all species, log-transformed relative abundances and Presence/Absence status used in the M-GWAS analysis. For all box plots, the central line, box and whiskers represent the median, interquartile range (IQR) and 1.5 times the IQR, respectively. The outliers were also been shown.

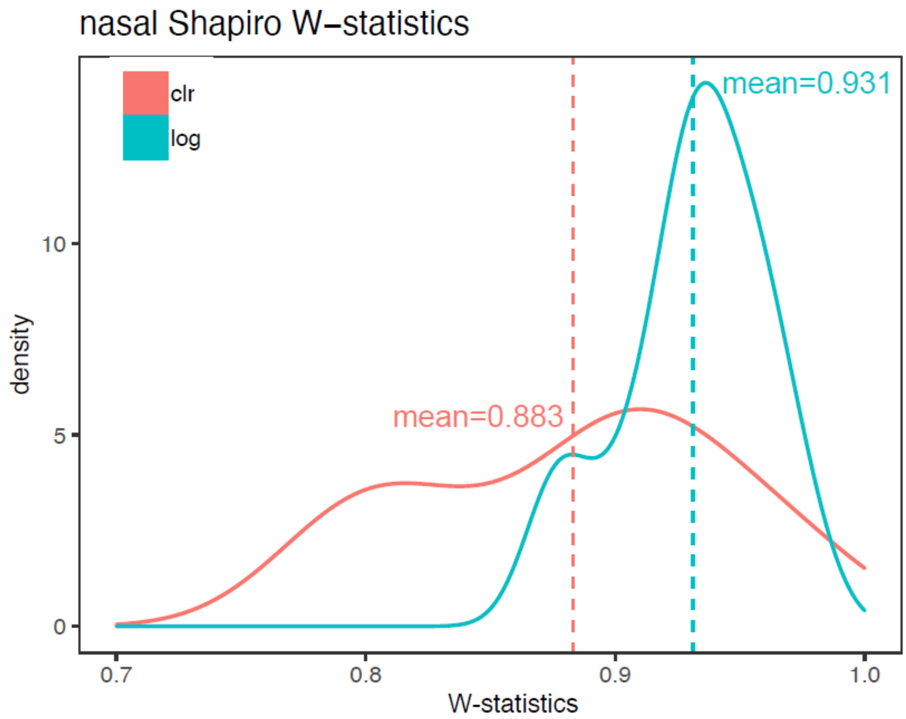

Supplementary Fig. 20. Estimation of the centered-log transformed (CLR) and log-transformed W-statistics.

Density plot of the Shapiro W-statistics for CLR (red) and log-transformed (green) abundance data for 43 taxa/pathways entering the linear regression analysis. The log-transformed data conformed more strongly to normality with more GWAS tests close to 1.
