# Supplementary material for: A genome-wide association study reveals the relationship between human genetic variation and the nasal microbiome
Source: Commun Biol. 2024 Jan 30;7:139. doi: 10.1038/s42003-024-05822-5 (PMC10828421; doi:10.1038/s42003-024-05822-5)
Supplement: Supplementary file 3 — Description of Additional Supplementary Files [file 42003_2024_5822_MOESM3_ESM.pdf]

Description of Additional Supplementary Files

File name: Supplementary Data 1
Description: Data production summary.

File name: Supplementary Data 2
Description: The correlations of taxa with host PC1 and their difference between the northern and southern Chinese.

File name: Supplementary Data 3
Description: Host genetic variants associated with alpha diversity and the top 10 PcoAs.

File name: Supplementary Data 4
Description: 293 independent nasal microbial features (86 microbial taxa and 207 functions) used for analysis.

File name: Supplementary Data 5
Description: Nasal M-GWAS identified 180 independent genome-wide significant associations ($p < 5 \times 10$-8).

File name: Supplementary Data 6
Description: The variance explained for taxa and pathways using the 63 top loci.

File name: Supplementary Data 7
Description: The 33 top genes and their differentially expressed p-value in the 50 tissues (-log10(pvalue) were listed in the cells).

File name: Supplementary Data 8
Description: The associations of nasal microbial taxa and host genetic variants with suggestive $p<10^{-6}$.

File name: Supplementary Data 9
Description: Top 21 genetic variants associated with beta diversity (p<1e-5).

File name: Supplementary Data 10
Description: 45 host factors significantly associated with β-diversity (BH-adjusted FDR < 0.05), via PERMANOVA analysis.

File name: Supplementary Data 11
Description: The significant observational correlations between host traits (mainly metabolites) and nasal microbiome features (FDR adjusted $P < 0.05$).

File name: Supplementary Data 12
Description: One-sample BMR identifying 4 Bonferroni-corrected (128 suggestive) significant causal relationships between microbiome features and host traits (mainly metabolites) after multiple test correction ($p < 1.09 \times 10^{-4}$).

File name: Supplementary Data 13
Description: The concordance of WGS and integrated WGS.

File name: Supplementary Data 14
Description: Effect of adding more covariates on genome-wide significant results.

File name: Supplementary Data 15
Description: Source Data for the Fig. 3e-f.
