# Supplementary material for: A genome-wide association study reveals the relationship between human genetic variation and the nasal microbiome
Source: Commun Biol. 2024 Jan 30;7:139. doi: 10.1038/s42003-024-05822-5 (PMC10828421; doi:10.1038/s42003-024-05822-5)
Supplement: Supplementary file 5 — Reporting Summary [file 42003_2024_5822_MOESM5_ESM.pdf]

nature portfolio

Corresponding author(s): Tao Zhang

Last updated by author(s): Jan 5, 2024

# Reporting Summary

Nature Portfolio wishes to improve the reproducibility of the work that we publish. This form provides structure for consistency and transparency in reporting. For further information on Nature Portfolio policies, see our Editorial Policies and the Editorial Policy Checklist.

## Statistics

For all statistical analyses, confirm that the following items are present in the figure legend, table legend, main text, or Methods section.

| n/a | Confirmed | |
|---|---|---|
| ☐ | ☒ | The exact sample size (*n*) for each experimental group/condition, given as a discrete number and unit of measurement |
| ☐ | ☒ | A statement on whether measurements were taken from distinct samples or whether the same sample was measured repeatedly |
| ☐ | ☒ | The statistical test(s) used AND whether they are one- or two-sided<br>*Only common tests should be described solely by name; describe more complex techniques in the Methods section.* |
| ☐ | ☒ | A description of all covariates tested |
| ☐ | ☒ | A description of any assumptions or corrections, such as tests of normality and adjustment for multiple comparisons |
| ☐ | ☒ | A full description of the statistical parameters including central tendency (e.g. means) or other basic estimates (e.g. regression coefficient) AND variation (e.g. standard deviation) or associated estimates of uncertainty (e.g. confidence intervals) |
| ☐ | ☒ | For null hypothesis testing, the test statistic (e.g. *F*, *t*, *r*) with confidence intervals, effect sizes, degrees of freedom and *P* value noted<br>*Give P values as exact values whenever suitable.* |
| ☒ | ☐ | For Bayesian analysis, information on the choice of priors and Markov chain Monte Carlo settings |
| ☐ | ☒ | For hierarchical and complex designs, identification of the appropriate level for tests and full reporting of outcomes |
| ☐ | ☒ | Estimates of effect sizes (e.g. Cohen's *d*, Pearson's *r*), indicating how they were calculated |

*Our web collection on statistics for biologists contains articles on many of the points above.*

## Software and code

Policy information about availability of computer code

| | |
|---|---|
| Data collection | For all 4D-SZ individuals, an extensive dataset of measured and self-reported phenotypic information, as well as blood and stool samples, had been collected during physical examination, as previously reported(doi:10.1038/s41421-021-00356-0,doi:10.1038/s41588-021-00968-y et al) |
| Data analysis | BWA software(version 0.7.15)<br>Samtools software(version 0.1.18)<br>Picardtools software(version 1.62)<br>The Genome Analysis Toolkit software(GATK, version 3.8)<br>BEAGLE software(version 5.0)<br>SOAP software(version 2.22)<br>R software(version 3.2.5)<br>GCTA software(version 1.26)<br>PLINK software(version 1.90)<br>One-sample MR (TSLS method in R package)<br>MetaPhlAn3(version 3.0.7)<br>HUMAnN3(version 3.0.0) |

For manuscripts utilizing custom algorithms or software that are central to the research but not yet described in published literature, software must be made available to editors and reviewers. We strongly encourage code deposition in a community repository (e.g. GitHub). See the Nature Portfolio guidelines for submitting code & software for further information.

## Data

Policy information about availability of data

All manuscripts must include a data availability statement. This statement should provide the following information, where applicable:

- Accession codes, unique identifiers, or web links for publicly available datasets
- A description of any restrictions on data availability
- For clinical datasets or third party data, please ensure that the statement adheres to our policy

The data in this study have been deposited to GSA with the project id: PRJCA015657 (https://ngdc.cncb.ac.cn/bioproject/browse/PRJCA015657). All GWAS summary statistics data that support the findings of this study including associations between host genetics and nasal microbiome are publicly available in https://ngdc.cncb.ac.cn/gvm/getProjectFile?t=9f187d05 (access id: GVP000013). The nasal metagenomic sequencing data after removing host reads in this study have been deposited to GSA and available in https://ngdc.cncb.ac.cn/gsa-human/browse/HRA004206 (access id: HRA004206). The release of these data was approved by the Ministry of Science and Technology of China (Project ID: 2023BAT0694). According to the Human Genetic Resources Administration of China regulation and the institutional review board of BGI-Shenzhen related to protecting individual privacy, the human blood sequencing data are controlled-access and are available via an application on request.

## Human research participants

Policy information about studies involving human research participants and Sex and Gender in Research.

| | |
|---|---|
| Reporting on sex and gender | in this study we used self-reported sex information and 63% were females. |
| Population characteristics | For all 4D-SZ individuals, an extensive dataset of measured and self-reported phenotypic information, as well as blood and stool samples, had been collected during physical examination, as previously reported(doi:10.1038/s41421-021-00356-0,doi:10.1038/s41588-021-00968-y et al) |
| Recruitment | Individuals were recruited independently of this study, and was based on voluntary participation after an invitation letter. |
| Ethics oversight | All participants provided informed written consent, and all study procedures were performed in accordance with the World Medical Association Declaration of Helsinki ethical principles for medical research. |

Note that full information on the approval of the study protocol must also be provided in the manuscript.

# Field-specific reporting

Please select the one below that is the best fit for your research. If you are not sure, read the appropriate sections before making your selection.

☒ Life sciences ☐ Behavioural & social sciences ☐ Ecological, evolutionary & environmental sciences

For a reference copy of the document with all sections, see nature.com/documents/nr-reporting-summary-flat.pdf

# Life sciences study design

All studies must disclose on these points even when the disclosure is negative.

| | |
|---|---|
| Sample size | In this study, 1,593 nasal samples from the cohort were collected for whole metagenomic sequencing in 2018 (Supplementary Table 1). 1,457 of the 1,593 individuals also had blood samples with whole genome sequencing. |
| Data exclusions | We filtered variants to meet these thresholds: (i) Hardy-Weinberg equilibrium (HWE) p > 10-6; and (ii) genotype calling rate > 98%. We demanded samples to meet these criteria: (i) mean sequencing depth > 5×; (ii) variant calling rate > 98%; (iii) no population stratification by performing principal components analysis (PCA) analysis implemented in PLINK92 (v1.9) and (iv) excluding related individuals by calculating pairwise identity by descent (IBD, Pi-hat threshold of 0.1875) in PLINK. After variant and sample quality control, 1,401 individuals with about 7 million common and low-frequency (MAF ≥ 1%) variants were left for M-GWAS analyses. |
| Replication | We have no replication cohort.The limitations has been described in discussions in main text. |
| Randomization | This is not an expertimental study. Randomization is not applicable |
| Blinding | This is not an experimental study. Blinding is not applicable |

# Reporting for specific materials, systems and methods

We require information from authors about some types of materials, experimental systems and methods used in many studies. Here, indicate whether each material, system or method listed is relevant to your study. If you are not sure if a list item applies to your research, read the appropriate section before selecting a response.

## Materials & experimental systems

| n/a | Involved in the study |
| --- | --- |
| ☒ | ☐ Antibodies |
| ☒ | ☐ Eukaryotic cell lines |
| ☒ | ☐ Palaeontology and archaeology |
| ☒ | ☐ Animals and other organisms |
| ☒ | ☐ Clinical data |
| ☒ | ☐ Dual use research of concern |

## Methods

| n/a | Involved in the study |
| --- | --- |
| ☒ | ☐ ChIP-seq |
| ☒ | ☐ Flow cytometry |
| ☒ | ☐ MRI-based neuroimaging |
